# Risk Factors and Hospitalizations Associated with Pediatric Adenovirus and Rotavirus Infections in Northern Lebanon

**DOI:** 10.3390/medicina61020296

**Published:** 2025-02-08

**Authors:** Sara Khalife, Marwan Osman, Sara Moubayed, Issmat I. Kassem, Dima El Safadi

**Affiliations:** 1 Department of Medical Laboratory Technology, Faculty of Health Sciences, Beirut Arab University, Tripoli 11-5020, Lebanon; sara.khalifeh@bau.edu.lb (S.K.); sarah.moubayed@hotmail.com (S.M.); 2 Department of Neurosurgery, Yale University School of Medicine, New Haven, CT 06510, USA; 3 Center for Food Safety, Department of Food Science and Technology, University of Georgia, 1109 Experiment Street, Griffin, GA 30223-1797, USA; issmat.kassem@uga.edu; 4 Faculty of Agricultural and Food Sciences, American University of Beirut, Riad El Solh, Beirut 1107-2020, Lebanon; 5 Department of Clinical Sciences, Liverpool School of Tropical Medicine, Liverpool L7 8XZ, UK; dima.elsafadi@lstmed.ac.uk

**Keywords:** acute gastroenteritis, rotavirus, adenovirus, pediatric infections, risk factors, clean water, North Lebanon

## Abstract

*Background and Objectives*: Acute gastroenteritis (AGE) is a major contributor to pediatric morbidity and mortality worldwide. There is a scarcity of data on AGE in North Lebanon, a region profoundly affected by the Syrian refugee crisis and water sanitation issues. This study examines the prevalence, risk factors, and seasonal variations in adenovirus and rotavirus infections in children with AGE in North Lebanon. *Materials and Methods*: A cross-sectional study was conducted from September 2022 to August 2023 on 400 children (1 month to 5 years old) with AGE that were admitted to pediatric departments of three private hospitals in North Lebanon. Stool samples were collected and tested using chromatographic immunoassays. Comprehensive demographic and clinical data were collected and analyzed. *Results*: Rotavirus was the most frequent viral agent (28%), followed by adenovirus (12.3%) and mixed infections (5.5%). Rotavirus vaccination demonstrated a significant protective effect, and lower infection rates were associated with breastfeeding and consumption of bottled water (*p* < 0.001). Higher infection rates correlated with lower levels of maternal education and household incomes (*p* < 0.001). Malnutrition significantly increased susceptibility to rotavirus infections (*p* < 0.001). *Conclusions*: This study emphasized the urgent need for targeted public health interventions in North Lebanon to mitigate the burden of rotavirus and adenovirus-induced acute gastroenteritis among children.

## 1. Introduction

Acute gastroenteritis (AGE) is a significant contributor to morbidity and mortality worldwide and predominantly impacts children under the age of five years [1]. Three to five billion gastroenteritis cases were reported among hospitalized children globally, with a death rate of 1.5 to 2.5 million annually [2]. AGE-associated mortality rates are predicted to be significantly higher in low-income countries with limited healthcare services and hygienic measures [3]. In comparison, mortality rates have decreased considerably in industrialized regions, but morbidity has persisted, posing serious clinical and economic challenges [4].

AGE cases typically manifest with mild symptoms, including a low consistency of stool (watery diarrhea), an increase in the frequency of excretion (≥3 in 24 h), and fever or vomiting, lasting less than one week. More severe cases with extended clinical symptoms can require hospitalization and microbiological examinations [5]. Diarrheal episodes, a major symptom of AGE, are estimated to cause approximately 2.5 million deaths annually in children under 5 years old, particularly in tropical regions [6].

Acute gastroenteritis can be transmitted among susceptible individuals through the fecal–oral route and via contaminated surfaces and water resources [7]. In general, viruses, the major etiological agents of AGE, are responsible for 70–90% of positive cases in children. AGE-causing viruses include rotavirus (RV) and enteric adenovirus (AdV) [8]. Group A rotavirus (RVA) is considered the predominant cause of severe childhood diarrhea [8,9].

Approximately 111 million cases of rotavirus infections are recorded annually among children, resulting in a death rate of 400,000 individuals and 2 million cases of hospitalization in Asian and African countries [10]. An estimated 82% of RVA-associated deaths in developing countries are linked to malnutrition or lack of access to adequate and safe rehydration [9]. Human Adenoviruses (HAdVs), particularly serotypes 40 and 41, are also implicated in childhood gastroenteritis. HAdVs can cause a spectrum of symptoms beyond gastroenteritis, including respiratory illness, conjunctivitis, and exanthema [11].

Incidences of rotavirus and adenovirus infections among hospitalized children have been reported across the globe in different epidemiological studies, which highlighted the critical need to implement effective prevention measures to halt the global spread of viral gastroenteritis [12,13,14,15,16,17,18,19]. Studies on viral gastroenteritis epidemiology are generally limited in the Middle East and North Africa (MENA) region, which comprises 21 countries or territories, several of which have experienced instability, war, natural disasters, and/or economic and infrastructure challenges in the recent past. However, a previous report analyzing viral gastroenteritis rates in the MENA region found the lowest mean prevalence of rotavirus in Qatar (14%), followed by Sudan (18%), and Tunisia (20%). In comparison, the highest prevalence was observed in Oman (47%), followed by Libya (41%), and Jordan (37%) [20]. Adenovirus infections were primarily detected in the UAE and Iraq, with a mean rate of 13% and 12%, respectively [20]. Caution should be taken when interpreting these studies as different diagnostic methods were used.

Lebanon, a MENA country with a well-documented plethora of public health and economic challenges, has a hybrid healthcare system comprising public and private sectors. The Ministry of Public Health (MOPH) oversees public health services, including disease prevention initiatives and hospital regulation, but limited funding constrains its capabilities. However, the private sector, comprising hospitals, clinics, and specialized medical centers, plays an important role in healthcare delivery, particularly in urban areas. These facilities provide high-quality medical services and attract both local and international patients, yet the high cost of care poses significant affordability concerns, particularly for uninsured populations. Non-governmental organizations (NGOs) also contribute to healthcare, particularly in underdeveloped areas and during humanitarian crises. Despite Lebanon’s strong medical education and research capacity, the healthcare system remains strained by financial instability, rural accessibility gaps, and reliance on external aid.

Limited research exists on viral gastroenteritis in Lebanese pediatric populations. Specifically, a prior study in Southern Lebanon revealed rotavirus (66.2%, 204/308) and adenovirus (25.3%, 78/308) to be the predominant pathogens in children with AGE, with co-infections present in 8.4% (26/308) of cases [21]. Another study in South Lebanon identified *Entamoeba histolytica* as the predominant parasitic agent (27.8%, 172/619) in hospitalized children. Notably, rotavirus, adenovirus, and other co-pathogens were also detected with a prevalence of 13.6% (84/619), 6.1% (38/619), and 6.1% (38/619), respectively [22]. To our knowledge, only one study conducted in North Lebanon in 2010 screened for enteric viral agents among hospitalized children, finding rotavirus and norovirus genogroup II with prevalences of 48% (38/79) and 6% (5/79), respectively [23]. However, North Lebanon is considered one of the most economically challenged parts of Lebanon, with populations lacking access to proper medical services, safe water and food, and hygienic tools [24,25,26]. To compound these challenges, this region has been receiving hundreds of thousands of refugees fleeing the protracted Syrian war across the border since 2011. Furthermore, water contamination issues and diarrheic illnesses in North Lebanon have been continuously and anecdotally reported in the media. Taken together, it is clear that data from 2010 on enteric viral agents among hospitalized children in the North might underestimate the current burden associated with these pathogens. Therefore, contemporary epidemiological surveillance in North Lebanon is crucial to understand viral circulation, clinical features, and risk factors for pediatric viral infections. This information is vital for healthcare professionals to implement effective infection control and vaccination strategies, particularly in regions facing water and hygiene challenges. Therefore, this study aims to determine the prevalence of rotavirus and adenovirus infections among children in hospitals in North Lebanon, along with identifying associated risks.

## 2. Materials and Methods

### 2.1. Study Design

A cross-sectional study was carried out between September 2022 and August 2023 in three private hospitals located in Tripoli and Al-Koura in North Lebanon. These hospitals serve a population ranging from 350,000 to 500,000 people, representing around 29–42% of the total population of North Lebanon. The socio-political and environmental conditions remained relatively stable throughout the study period, with no exceptional weather events observed that could have substantially influenced the data.

### 2.2. Study Population

Children 1 month to 5 years old, who were admitted to the hospitals with acute gastroenteritis (AGE) or diarrhea, defined as three or more loose or liquid stools within a day [5], were included in the study. Non-Lebanese children as well as those with chronic diarrhea or immunodeficiency were excluded from this study.

### 2.3. Sample Size Estimation

This study has a 95% confidence level with an alpha error of 0.05. The sample size was calculated using the prevalence estimation formula based on a prior estimate of 40.4% for the prevalence of AGE among inpatients in South Lebanon [2]. The study’s power settled at 90%, with a beta error of 0.10. Based on these calculations, the required sample size was 400 children. Therefore, a total of 400 stool specimens were collected from 400 children with AGE admitted to three private hospitals in North Lebanon.

### 2.4. Data and Sample Collection

Fresh stool samples were provided from study participants, labeled, placed on ice packs in a cool box, and transported under aseptic techniques to the biomedical laboratory of the Beirut Arab University for further analysis. Stool samples were tested within less than an hour for the presence of infectious agents using the rotavirus and adenovirus kit tests (CerTest; Biotec, Zaragoza, Spain) as previously described [21].

The focus of the study was on these pathogens, which are known to be among the most prevalent viral pathogens causing pediatric gastroenteritis in North Lebanon, with a higher likelihood of hospitalization compared to caliciviruses [23].

As per the manufacturer’s specifications, the diagnostic test used in this study demonstrated a sensitivity > 99%, specificity > 98%, a positive predictive value (PPV) > 94%, and a negative predictive value (NPV) > 99%, indicating its high accuracy and reliability for the detection of adenovirus and rotavirus infections.

For each individual included in the study, sociodemographic, clinical data, and vaccination history were collected using a standardized questionnaire. The Vesikari Score System was used for the calculation of the clinical severity index [27].

### 2.5. Statistical Analysis

Statistical analyses were performed using IBM Statistical Packages for Social Sciences (IBM SPSS, version 22.00, IBM Corp, Armonk, NY, USA). Chi-square or Fisher’s exact tests were used to assess the differences between variables. *p* values of ˂0.05 were considered significant. Odds ratios (ORs) with 95% confidence intervals (95% CIs) were used to determine the association of variables with adenovirus and rotavirus infections. Bivariable and multivariable logistic regression analyses were used to determine the factors associated with adenovirus and rotavirus infections. All factors with a *p*-value of <0.05 on the univariate analysis were subjected to the multivariable analysis.

### 2.6. Ethical Consideration

The study was conducted according to the guidelines and regulations of the World Medical Association’s Code of Ethics (Declaration of Helsinki). The study was approved by the Institutional Review Board (IRB) of the Beirut Arab University (IRB number: 2023-H-0146-HS-R-0517). Written informed consent was obtained from parents or legal guardians.

## 3. Results

### 3.1. Sociodemographic Characteristics of the Study Participants

Among the 400 children that were included in this study, 58.5% (234/400) were males and 41.5% (166/400) were females. Patients were categorized into six age groups, spanning 1 to 60 months (1–3, 4–11, 12–23, 24–35, 36–47, and 48–60 months) to investigate potential associations with disease severity and occurrence as previously described [28]. The mean family size of patients was 3.81, while 60% (240/400) of participants were breastfed and 74% (296/400) had received the rotavirus vaccination (Table 1).

Notably, 31.5% (126/400) of patients’ guardians/mothers reported no formal education, while 27.8% (111/400) had completed primary education and 27.3% (109/400) and 13.5% (54/400) had secondary and tertiary levels of education, respectively. Furthermore, 55% (220/400) of the patients resided in urban areas, while 45% (180/400) were located in rural regions. Based on Lebanese criteria, following the country’s economic crisis, a significant shift in household income distribution was observed. An analysis of household income distribution revealed that most patients (54.5%, 218/400) fell within the medium income category, while 31.3% (125/400) and 14.3% (57/400) reported low and high household incomes, respectively. It is important to note that, due to the economic crisis, the Lebanese pound lost more than 90% of its value in 2019. As a result, the lowest income is now equivalent to approximately USD 100 per month, while the highest income is around USD 2000–USD 5000 per month in certain multinational companies or high-end job sectors [29]. Over half of the patients (56.3%, 225/400) reported contact with domestic animals. In terms of water consumption, mineral bottled water was the preferred source for the majority of the patients (65%, 260/400), while 35% (140/400) relied on tap water. The nutritional status assessment revealed that 80.5% (322/400) of patients exhibited no signs of malnutrition, while 8.3% (33/400) and 11.3% (45/400) displayed mild and moderate malnutrition, respectively (Table 1).

Rotavirus was detected in the stool samples of 112 patients, accounting for 28% of the cases. Adenovirus was found in 49 cases (12.3%), while mixed infections with both rotavirus and adenovirus were observed in 22 cases (5.5%) (Table 1). The analysis of age distribution across the three types of infections (rotavirus, adenovirus, and mixed infections) revealed significant differences (Table 1). Notably, the majority of rotavirus-infected patients were 12–23 months old (35.7%) and 24–35 months old (33.3%). In contrast, adenovirus-infected patients were predominantly between 4 and 11 months (16.7%) and 24 and 35 months (14.8%) of age. Patients co-infected with both viruses were mainly between 4 and 11 months old (10%) (*p* = 0.02).

No significant associations were observed between sex, geographical location, contact with domestic animals, and the three types of infections. However, a significant association was found with rotavirus vaccination status (*p* < 0.001) (Table 1). Notably, the majority of rotavirus-infected patients (69.2%) and those co-infected with rotavirus/adenovirus (9.6%) had not received the rotavirus vaccine. Furthermore, breastfed patients and those who consumed mineral or bottled water showed the lowest infection rates (*p* < 0.001) (Table 1). In contrast, patients whose mothers or guardians lacked formal education and those from low-income households showcased the highest prevalence of infections (*p* < 0.001) (Table 1).

### 3.2. Clinical Characteristics of Study Participants

Table 2 details comprehensive clinical profiles of the patients across the three infection types (rotavirus, adenovirus, and mixed infections). Fever was the most prevalent symptom observed in 32.8% of the patients, followed by diarrhea at 29.5% and vomiting at 22.5%. Fever exhibited a significantly higher occurrence in both the rotavirus and mixed infection groups (75.9% and 77.3%, respectively) compared to the adenovirus group (59.2%) (*p* = 0.001). Vomiting and diarrhea were notably more prevalent in the mixed infection group (*p* < 0.001 and *p* = 0.001, respectively). Furthermore, significant differences were observed in the mean Vesikari scores among the three infection types (*p* < 0.05), indicating varying severity levels of gastroenteritis. Additionally, significant differences were noted in the duration of hospitalization across the infection types (*p* = 0.02) (Table 2).

### 3.3. Monthly Distribution of Rotavirus and Adenovirus Infections

**Figure 1:**
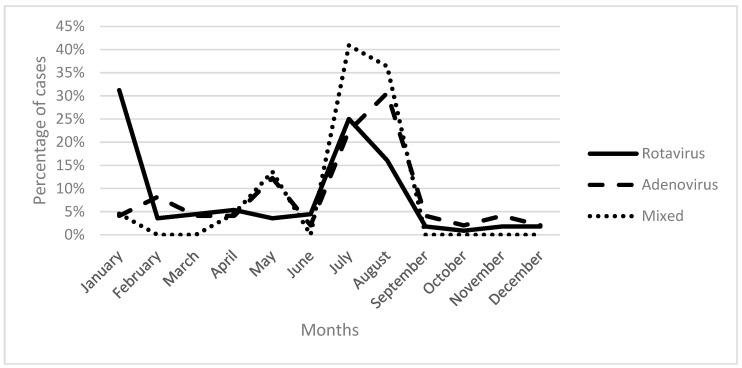
Monthly prevalence of adenovirus and rotavirus infections in children in North Lebanon.

Rotavirus infection exhibited a consistent presence throughout the year, with the majority of cases occurring in January (31.25%) and July (25%). In comparison, adenovirus and mixed infections peaked in July and August (Figure 1).

### 3.4. Multivariable Logistic Regression Analysis

Our analysis showed that breastfeeding had a potential protective effect against rotavirus, adenovirus, and mixed infections, suggesting its beneficial impact on reducing susceptibility to these pathogens (*p* < 0.05) (Table 3). Additionally, the administration of the rotavirus vaccine exhibited significant protection specifically against rotavirus infection, highlighting the efficacy of this vaccination in preventing targeted pathogens (*p* < 0.001) (Table 3). Our analysis also revealed that access to clean water was associated with a reduced frequency of rotavirus, adenovirus, and mixed infections, underscoring the importance of sanitation in mitigating the risk of infections (*p* < 0.001). A noteworthy observation from our study is the elevated risk of rotavirus infections among patients whose guardians lack formal education (*p* < 0.05), highlighting the importance of addressing educational disparities in public health strategies aimed at reducing the burden of infectious diseases (Table 3). Furthermore, our analysis revealed a significant correlation between nutritional status and infection risk. Children with mild or moderate malnutrition had a higher risk of rotavirus infections (*p* < 0.001), emphasizing the critical role of nutritional status in modulating immune response and disease resistance, particularly against rotavirus (Table 3).

Age-related susceptibility was evident, with rotavirus infections significantly higher among 12 to 23 months old children (*p* < 0.05) in comparison to other age groups. Adenovirus and mixed infections followed a similar age distribution pattern across the studied age groups.

## 4. Discussion

Epidemiological surveillance of AGE among hospitalized children is critically important in Lebanon. This provides health practitioners and other stakeholders with a suitable understanding, allowing them to uncover the incidence rates, clinical manifestations, and potential risk factors associated with these infections, which will contribute to developing preventive strategies to halt the spread of AGE in pediatric populations.

In this study, out of 400 hospitalized children with AGE, 45.8% (183/400) tested positive for adenovirus and/or rotavirus infections. Rotavirus infections had a prevalence of 28% (112/400), which was higher than that of adenovirus infections at 12.3% (49/400) and mixed infections at 5.5% (22/400). Our findings are consistent with a previous study conducted in North Lebanon, which assessed the prevalence and clinical characteristics of enteric pathogens in patients with acute community-acquired diarrhea and reported a prevalence of rotavirus of 27.5% (27/360) and a prevalence of adenovirus of 5% (18/360), indicating consistency with regional data on enteric pathogens [24]. A previous study conducted in South Lebanon reported a comparable prevalence of single pathogen infections and mixed infections among hospitalized children at levels of 47.5% (294/619) and 6.1% (38/619), respectively [22]. However, another study in South Lebanon demonstrated higher infection rates for rotavirus at 66.2% (204/308) and/or adenovirus at 25.3% (78/308) in comparison to our findings [21].

In Iraq (another MENA country), the incidence of rotavirus infections among hospitalized children closely matches our study, with an infection rate of 28.1% (295/1050). However, the prevalence of adenovirus infections is slightly higher at 17.1% (179/1050) [19]. Conversely, a study in Riyadh, Saudi Arabia, reported a lower prevalence of rotavirus and adenovirus infections at 2% (2/100) and 7% (7/100), respectively [30]. This contradicts our findings and those of other surveillance studies, which have identified rotavirus as the most prevalent enteric pathogen among the examined patients. However, it should be noted that the economic conditions in Saudi Arabia are among the highest in the MENA region. Additionally, several other factors might contribute to the observed heterogeneity in the prevalence of enteric pathogens between the aforementioned studies, including geographical location, diagnostic techniques, and sample size [30].

In our study, regression analysis revealed that the age group of 12–23 months is significantly associated with an increased risk of acquiring rotavirus infections among hospitalized children (OR = 2.53, 95% CI [1.01–6.35], *p* = 0.04) (Table 3). This is consistent with several reports from Lebanon, Mozambique, China, Myanmar, and Pakistan [15,21,31,32,33]. Within this age group, the acquisition of rotavirus infections is likely facilitated by fecal–oral transmission, exposure to contaminated water sources used for formula preparation, and/or routine hygiene practices [21]. An analysis of additional demographic factors demonstrated a significant association (*p* < 0.05) between non-breastfeeding, lack of rotavirus vaccination, maternal/guardian illiteracy, low socioeconomic status, tap water consumption, and moderate malnutrition and an increased prevalence of rotavirus and mixed infections (Table 1). A similar trend was observed for adenovirus, with the exception that mild nutrition was associated with a higher infection rate (Table 1). Interestingly, previous studies in Lebanon have highlighted notable microbial contamination of water and food in North Lebanon [24,26].

Breastfeeding conferred a protective effect against rotavirus, adenovirus, and mixed infections compared to the non-breastfed group (*p* < 0.05) (Table 3). In comparison, a previous study from South Lebanon did not observe a statistically significant association between breastfeeding and reduced rotavirus incidence, but it suggested a protective role of breastfeeding against adenovirus and mixed infections, which aligns with our regression analysis [22]. Taken together, these findings highlight the critical importance of promoting breastfeeding practices. It has been established that breast milk can provide immunological and nutritional components that serve as an effective preventive measure against childhood diarrhea [34].

In our study, there was a statistically significant association between rotavirus vaccination and a reduced incidence of rotavirus infection (OR = 14.40, 95% CI [8.44–24.54], *p* < 0.0001) (Table 3). This aligns with previous research conducted in Lebanon, which demonstrated the vaccine’s effectiveness in preventing rotavirus infections [21,22]. Notably, several countries, have recently included rotavirus vaccination in their national immunization programs, including Lebanon, the Netherlands, India, the Democratic Republic of Congo, and Afghanistan [35,36,37]. Additionally, our study showed that consuming bottled mineral water significantly protected against all three enteric pathogen groups in pediatric patients. However, factors such as maternal/guardian illiteracy and moderate/mild malnutrition were identified as significant risk factors for rotavirus infections (Table 3).

In our study, patients with mixed infections experienced significantly higher frequencies of fever, vomiting, and diarrhea compared to those infected with adenovirus or rotavirus alone (*p* < 0.05). Fever was the most prevalent symptom observed in single-pathogen infections, with a reported prevalence of 75.89% (85/112) for rotavirus and 59.18% (29/49) for adenovirus (Table 2). These findings differ from a study conducted in Indonesia, which identified vomiting as the predominant clinical manifestation of rotavirus infections [38]. Similarly, research from Peru reported a higher prevalence of vomiting compared to other symptoms in both viral mono-infection and co-infection cases [14]. These differences highlight the importance of regional epidemiological studies, because they can account for several unique factors related to the population, geographic location, and specific socioeconomic conditions.

Our study highlighted the potential severity of co-infections with enteric pathogens in pediatric patients with acute gastroenteritis. Patients with mixed infections exhibited significantly higher Vesikari scores (*p* = 0.04) and longer hospital stays (*p* = 0.02) compared to those with single viral infections (Table 2). It is important to note that a previous study from Lebanon identified *E. histolytica* infection as the cause of severe clinical presentation with a Vesikari score of 11.8 ± 1.6 [22]. These findings suggest that *E. histolytica* infection might lead to a harsher clinical course compared to viral mono-infections and viral co-infections in Lebanon. Conversely, findings reported by Zaraket et al. (2020) support our results regarding the severity of mixed viral infections and reveal a significant prevalence of high-grade fevers in the mixed infection group (*p* < 0.003) [21]. These findings highlight the need for further research to reveal the specific contributions of different enteric pathogens to disease severity.

In our study, rotavirus was more prevalent in January, accounting for 31.3% of cases, followed by additional peaks in July (25%) and August (16.1%) (Figure 1). Similarly, a higher incidence of mixed infections was observed during July and August. This seasonal pattern of rotavirus is consistent with several reports from Lebanon, Iraq, Turkey, and Saudi Arabia [19,21,22,30,39]. Lower temperatures, humidity levels, and precipitation rates have been established as significant factors contributing to the environmental persistence and transmission of rotavirus. Consequently, weather conditions may lead to increased indoor occupancy, potentially facilitating the spread of the virus among susceptible individuals through contact with contaminated surfaces and objects [40]. Our study also reported a significant peak in adenovirus infections during August, reaching 30.6% of all cases. This summer seasonality in Lebanon may be attributable to factors such as inherent viral characteristics, including heat tolerance and environmental stability, as well as increased demand for water intake, which could potentially be contaminated [22]. These findings align with other studies conducted in Palermo (Sicily) and Lebanon [13,21,22]. However, research from Saudi Arabia presented contrasting results, demonstrating a high prevalence of adenovirus during autumn and early winter, particularly under colder temperatures [30]. This observed variation in adenovirus prevalence is likely due to geographical and climatic dissimilarities.

This study has several limitations. Firstly, the study timeframe could have been extended to collect more data on the clinical characteristics and risk factors associated with AGE in hospitalized children. Therefore, future research should involve larger-scale cohort studies with increased sample sizes. Another limitation is the absence of molecular examinations such as PCR and phylogenetic analysis. While chromatographic immunoassays are reliable and widely used for identifying adenovirus and rotavirus infections, they may be less sensitive than molecular methods like PCR, particularly in cases with low viral loads or atypical clinical presentations. Molecular techniques are crucial for ensuring high sensitivity and the specific detection of enteric pathogens during diagnosis. Moreover, identifying the predominant species and genotypes is essential for a clearer understanding of virus epidemiology and can guide clinicians to implement appropriate preventive measures to curb their spread within the Lebanese community. Despite this limitation, we believe that chromatographic immunoassays are still highly relevant within the scope of our study and can inform public health interventions and vaccination strategies.

Finally, the study did not include the detection of caliciviruses or bacterial or parasitic agents. Including these pathogens in future studies would be significant for assessing the severity and pathogenesis associated with both single-pathogen and mixed infections, providing a more holistic view of AGE etiology.

## 5. Conclusions

Despite the limitations, our study offers valuable insights. It provides essential baseline data on the prevalence and seasonal variations in adenovirus and rotavirus among children in North Lebanon, a region with unique socio-economic challenges. The comprehensive demographic and clinical data collected provide valuable insights into the epidemiology of AGE, which can inform public health strategies and clinical management practices. Additionally, the study highlights the positive impact of rotavirus vaccination, reinforcing the importance of vaccination programs. By identifying key areas for improvement in terms of diagnostic capabilities and preventive measures, this study lays a strong foundation for future studies and public health interventions aimed at reducing the burden of AGE in the region.

## Figures and Tables

**Table 1:** Socio-demographic and lifestyle characteristics of study participants.

Variables	Rotavirus n/N (%)	Adenovirus n/N (%)	Mixed Infections n/N (%)	N	*p*-Value
**Age (months)**					
1–3	9/40 (22.5%)	2/40 (5.0%)	0 (0.0%)	40	0.02 *
4–11	22/90 (24.44%)	15/90 (16.7%)	9/90 (10.0%)	90	
12–23	35/98 (35.71%)	9/98 (9.2%)	7/98 (7.1%)	98	
24–35	27/81 (33.33%)	12/81 (14.8%)	3/81 (3.7%)	81	
36–47	12/52 (23.07%)	6/52 (11.5%)	2/52 (3.8%)	52	
48–60	7/39 (17.94%)	5/39 (12.8%)	1/39 (2.6%)	39	
Total	112/400 (28%)	49/400 (12.3%)	22/400 (5.5%)	400	
**Sex**					
Male	70/234 (29.91%)	33/234 (22.5%)	13/234 (22.5%)	234	0.07
Female	42/166 (25.30%)	16/166 (22.5%)	9/166 (22.5%)	166	
Total	112	49	22	400	
**Location**					
Urban	65/220 (29.54%)	30/220 (13.6%)	9/220 (4.1%)	220	0.45
rural	47/180 (26.11%)	19/180 (10.6%)	13/180 (7.2%)	180	
Total	112	49	22	400	
**Breast feeding**					
No	78/160 (48.75%)	28/160 (17.5%)	14/160 (8.8%)	160	<0.0001 *
Yes	34/240 (14.16%)	21/240 (8.8%)	8/240 (3.3%)	240	
Total	112	49	22	400	
**Rotavirus vaccine**					
No	72/104 (69.23%)	22/104 (21.2%)	9/104 (9.6%)	104	<0.0001 *
Yes	40/296 (13.51%)	27/296 (9.1%)	13/296 (4.1%)	296	
Total	112	49	22	400	
**Mother/guardian level of education**					
No formal education	51/126 (40.47%)	20/126 (15.9%)	10/126 (7.9%)	126	<0.0001 *
Primary	33/111 (29.72%)	15/111 (13.5%)	6/111 (5.4%)	111	
Secondary	16/109 (14.67%)	8/109 (7.3%)	5/109 (4.6%)	109	
Tertiary	12/54 (22.22%)	6/54 (11.1%)	1/54 (1.9%)	54	
Total	112	49	22	400	
**Household income**					
Low	49/125 (39.2%)	17/125 (13.6%)	8/125 (6.4%)	125	<0.0005 *
Medium	45/218 (20.64%)	25/218 (11.5%)	12/218 (5.5%)	218	
High	18/57 (31.57%)	7/57 (12.28%)	2/57 (3.5%)	57	
Total	112	49	22	400	
Mean family size	3.6	3.5	3.7	3.81	<0.0001 *
**Contact with domestic animals**					
Yes	67/225 (29.77%)	27/225 (12%)	14/225 (6.2%)	225	0.30
No	45/175 (25.71%)	22/175 (12.6%)	8/175 (4.6%)	175	
Total	112	49	22	400	
**Source of drinking water**					
Mineral bottled water	45/260 (17.30%)	20/260 (7.7%)	6/260 (2.3%)	260	<0.0001 *
Tap water	67/140 (47.85%)	29/140 (20.7%)	16/140 (11.4%)	140	
Total	112	49	22	400	
**Weight/height score**					
Moderate malnutrition	34/45 (75.55%)	5/45 (11.1%)	5/45 (11.1%)	45	<0.0001 *
Mild malnutrition	22/33 (66.66%)	7/33 (21.2%)	2/33 (6.1%)	33	
No malnutrition	56/322 (17.39%)	37/322 (11.5%)	15/322 (4.7%)	322	
Total	112	49	22	400	

*: Statistically significant; N: number of patients with acute gastroenteritis.

**Table 2:** Clinical characteristics of enrolled patients.

Variables	Rotavirus	Adenovirus	Mixed Infections	Total	*p*-Value
Frequency (%)	112 (28%)	49 (12.3%)	22 (5.5%)	400	-
**Clinical manifestations**					
Fever	85 (75.9%)	29 (59.2%)	17 (77.3%)	131	0.001 *
Vomiting	54 (48.2%)	21 (42.9%)	15 (68.2%)	90	<0.0001 *
Diarrhea	73 (65.2%)	27 (55.1%)	18 (81.8%)	118	0.001 *
Vesikari score (Mean ± SD)	11.2 ± 1.2	11.02 ± 1.3	11.8 ± 1.6	-	0.04 *
Duration of hospital stay (days) (Mean ± SD)	2.4 ± 1.01	2.7 ± 1.2	3.1 ± 1.1	2.76 ± 1.3	0.02 *

*: Statistically significant; SD: Standard deviation.

**Table 3:** Factors associated with rotavirus, adenovirus, and mixed infections among North Lebanese children.

		Rotavirus		Adenovirus		Mixed Infections	
**Variables**		OR (95% CI)	*p* Value	OR (95% CI)	*p* Value	OR (95% CI)	*p* Value
Age groups (months)	1–3	1.25 (0.42–3.69)	0.68	0.35 (0.06–1.96)	0.23	0.31 (0.01–8.01)	0.48
	4–11	1.47 (0.57–3.81)	0.41	1.36 (0.45–4.04)	0.58	4.22 (0.51–34.53)	0.17
	12–23	2.53 (1.01–6.35)	0.04 *	0.68 (0.21–2.19)	0.52	2.92 (0.34–24.57)	0.32
	24–35	2.28 (0.89–5.84)	0.08	1.18 (0.38–3.62)	0.76	1.46 (0.14–14.52)	0.74
	36–47	1.37 (0.48–3.88)	0.55	0.88 (0.24–3.14)	0.85	1.52 (0.13–17.39)	0.73
	48–60						
Breastfeeding	No	5.76 (3.57–9.28)	<0.0001 *	2.21 (1.20–4.05)	0.01 *	2.78 (1.13–6.79)	0.02 *
	Yes						
Rotavirus vaccine	No	14.40 (8.44–24.54)	<0.0001 *	2.67 (1.20–4.05)	0.001 *	2.06 (0.85–4.97)	0.10
	Yes						
Water source	Tap water	4.38 (2.76–6.95)	<0.0001 *	3.13 (1.69–5.78)	0.0003 *	5.46 (2.08–14.30)	0.0005 *
	Mineral bottled water						
Guardian level of education	No formal education	2.38 (1.14–4.95)	0.02 *	1.50 (0.57–3.99)	0.40	4.57 (0.57–36.61)	0.15
	Primary	1.48 (0.69–3.16)	0.31	1.25 (0.45–3.42)	0.66	3.03 (0.35–25.80)	0.31
	Secondary	0.60 (0.26–1.38)	0.23	0.63 (0.20–1.92)	0.42	2.54 (0.29–22.37)	0.39
	Tertiary						
Household income	Low	1.39 (0.71–2.71)	0.32	1.12 (0.43–2.88)	0.80	1.88 (0.38–9.15)	0.43
	Medium	0.56 (0.29–1.07)	0.08	0.92 (0.37–2.26)	0.86	1.60 (0.34–7.37)	0.54
	High						
Nutritional status	Moderate malnutrition	14.68 (7.01–30.72)	<0.0001 *	0.96 (0.35–2.59)	0.94	2.55 (0.88–7.41)	0.08
	Mild malnutrition	9.5 (4.35–20.70)	<0.0001 *	2.07 (0.84–5.11)	0.11	1.32 (0.28–6.04)	0.72
	No malnutrition						

*: Statistically significant; OR: odds ratio; CI: confidence interval.

## Data Availability

The data presented in this study are available on request from the corresponding author because the type of information requires controlled access.

## Abbreviations

The following abbreviations are used in this manuscript:

AGE	Acute gastroenteritis
RV	Rotavirus
AdV	Enteric adenovirus
RVA	Group A rotavirus
HAdV	Human Adenoviruses
MENA	Middle East and North Africa
IRB	Institutional Review Board
MOPH	Ministry of Public Health
NGO	Non-governmental organizations
